# A fully automated drilling machine for printed circuit boards with superior path optimization

**DOI:** 10.1038/s41598-025-25707-9

**Published:** 2025-11-18

**Authors:** Mohamed Mamdouh, Ahmed Khalid, Reem Mahmoud, Osama Desouki, Sameh O. Abdellatif

**Affiliations:** The Electrical Engineering Department and FabLab, Centre of Emerging Learning Technologies CELT, British University in Egypt (BUE), Cairo, 11387 Egypt

**Keywords:** AI-driven automation, PCB manufacturing, Path planning, KiCAD EDA, G-Code generation, Electrical and electronic engineering, Software

## Abstract

This paper addresses a critical challenge in Printed Circuit Board (PCB) manufacturing by proposing an AI-driven, fully automated drilling machine that employs sophisticated path-planning techniques. Current methodologies often fail to adequately assess designs with varying hole sizes, diverse component placements, and complex geometries, leading to compromised precision and increased manufacturing times. Our innovative approach leverages advanced algorithms to intelligently analyze PCB designs and optimize drilling paths, significantly reducing production time and minimizing errors. By automating the drilling process, we enhance overall productivity while ensuring precise hole placement, essential for maintaining high-quality circuit boards. Utilizing KiCAD EDA software, we automate the generation of Gerber files and G-Codes, demonstrating a remarkable 13-fold improvement in routing efficiency compared to manual methods. Our case studies validate the effectiveness of this system, showcasing successful continuity tests and superior quality finishes in PCB production. This research contributes to the advancement of PCB manufacturing. It lays the groundwork for future explorations into machine learning algorithms and optimization techniques, further enhancing the efficiency and applicability of AI in electronics design.

## Introduction

Artificial intelligence (AI) is the intelligence machines use to behave like humans^1^. In particular, it is a field of computer science where machines can perceive, process, and analyze different types of signals and make reasonable decisions^2^. The purpose of AI is not to replace human thinking but to help humans in complex and repeated tasks to open the door for innovation^3^. AI has been used in a wide range of applications, from the first medical applications in the 1970 s, such as MYCIN^4^, to the PROSPECTOR^5^ application in mineral mining, which both used rule-based expert systems. However, the expert system was limited to the rules that comprised it and could not predict or use previous knowledge to draw conclusions. Later, fuzzy logic and machine learning techniques were introduced to overcome the shortages in expert systems. Today, AI has boosted the efficiency of several applications, including chatbots, search engines, and education^6^. Integrating AI into new applications might increase efficiency or product marketing, especially for applications that depend primarily on human interaction and decision-making^1,3,7–12^. However, the first steps of applying AI to the mentioned types of applications count for a small percentage of the required tasks. A good example of this type of application is the circuit design, fabrication, and characterization^8^.

In circuit design, the designer is responsible for all decisions in the design process, including component placement, routing, validation, and testing. AI is being used in circuit design to mainly alleviate the repetitive tasks of the designer and allow them to explore more complex tasks. There are software tools that help in this process, such as SnapMagic^13^, Flux AI^14^, and JITX^15^. In general, the current efforts in the circuit design field focus on simple tasks such as using reusable circuits from datasheets, placing a component, or finding a replacement for a part. The decision-making process is still for the designer in the circuit design process. In circuit fabrication, the advancement of technologies led to a revolution in various fields, and the concept of optimization became a driving point. Computer numerical control (CNC) optimization is essential as it becomes accessible. Additionally, CNC machines have high machining accuracy and precision in highly complicated parts. The AI algorithm includes Generic algorithms, Artificial neural networks, Artificial Immune Systems, Ant colony optimization, and particle swan optimization; each algorithm improves a specific aspect, starting with GA, where the manipulation of the solution is influenced by three main operators(crossover, reproduction, and mutation) which allow CNC software to solve optimization problems^16^, If the optimization power is accompanied by the ability to learn of the ANN where the ANN could study multiple approach of optimized path then processed to generate the best possible milling paths.

The robotic part is equally immersed in the working principle of the CNC machines, where the movement should be incremented at equivalent intervals. Additionally, the positioning of the head should be selected over repetitive positioning of the head. The Artificial immune system is an algorithm that is inspired by immune function^16^, where it optimizes the process by increasing the cutting and milling speed at specific positions, in addition to learning and adaptability, where it learns the dimensional positing and estimates distances that are reachable by the head without reposition developed in electronic circuit design to help electronic designers streamline the circuit design process. AI in circuit design allows innovation by automating ordinary and repetitive tasks like routing and component placement, thus allowing the designers to focus on more innovative aspects^17–19^. Additionally, AI may be used in design verification in a lab environment where the AI-powered system checks the circuit components’ compatibility before running and testing the design.

Various software tools have been involved in implementing AI in their EDA tools. EDA introduced an AI-based tool called SnapMagic, a copilot that adds intelligence to the circuit and makes the designer interact with it^13^. SnapMagic is trained on datasheets of the most used electronic parts. Thus, it has a memory of compatible parts that work under the same conditions and can be used to automate repetitive tasks to release the burden on the designer. Further, it can auto-complete circuit designs based on the official designs from the datasheets by adding recommendations to the designer^13^. SnapMagic facilitates interaction with the circuit board by allowing the designer to chat with it, like “Add an MCU with 2 SPI ports and USB 2.0 and connect the USB port to a USB-C connector.” Moreover, it can optimize the cost by recommending the replacement of expensive parts, thus reducing the entire bill of materials. The replacement is made based on the availability of the components from the local distributors and in the market in general^13^. Flux is an open cloud-based PCB design tool that, with the help of AI, can speed up the design of PCBs 10 times faster by automating repetitive tasks, making use of reusable designs, and synchronizing the work in a team environment. Flux can reduce the errors in the circuit design using real-time validation to discover the errors and correct them^14^. JITX is another software tool that uses simple codes to streamline the design process. Further, it offers testing and validation of the circuit components and generates a log file specifying the faults in the design, facilitating troubleshooting. It also controls supply chain distribution with the local suppliers to recommend component replacement, reduce design costs, and allow for efficient optimization^15^. JITX and SnapMagic co-parent are compatible with software tools like Altium, ca., dance, and Zuken; therefore, there is no need to change the already used tool^13,15^.

In the context of this paper’s exploration of the research gap and innovation, we address a critical challenge in PCB manufacturing: the need for enhanced drilling processes that harness artificial intelligence and advanced path-planning techniques. Current methodologies in the field frequently exhibit limitations in their capacity to comprehensively assess designs characterized by heterogeneous hole sizes, disparate component placements, and complex board geometries. This inadequacy not only jeopardizes the precision of hole placement—an essential factor for preserving the integrity of high-quality circuit boards—but also contributes to prolonged manufacturing times and elevated error rates due to suboptimal drilling route optimization. The research gap identified in the existing literature highlights the absence of integrated systems that effectively utilize AI-driven analysis for the optimization of drilling paths in PCB manufacturing. This study posits that the introduction of an intelligent system capable of analyzing PCB designs and determining the most efficient drilling trajectories can bridge this gap. The research problem is thus clearly defined: how can artificial intelligence and sophisticated path-planning algorithms be employed to enhance the efficiency and accuracy of the drilling process in PCB production?

To address this problem, we propose the following research hypothesis: the implementation of AI-driven path optimization algorithms will significantly reduce production time and minimize error rates in PCB drilling operations, thereby enhancing overall productivity and throughput while automating the drilling process to decrease manual intervention. This innovative approach is anticipated to not only improve quality control by ensuring precise hole placement but also to elevate the standard of circuit board quality. Furthermore, this research addresses the economic implications of PCB manufacturing by extending the lifespan of drilling equipment and reducing maintenance costs through an effective path design that mitigates tool wear. By systematically addressing these multifaceted challenges, this study aims to make a substantial contribution to the field of PCB manufacturing, ultimately fostering advancements that enhance both operational efficiency and product quality.

## Hardware setup

**Fig. 1:**
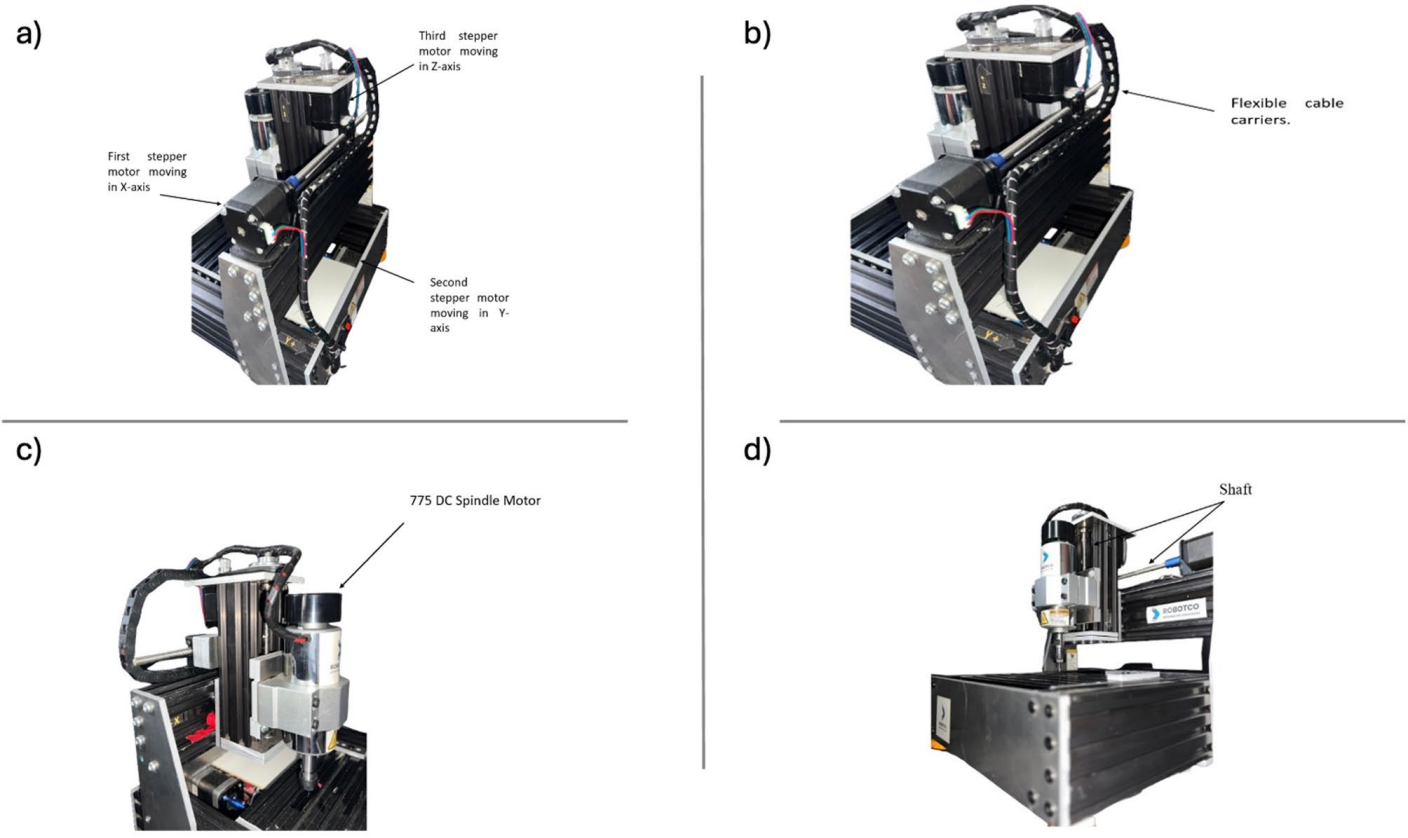
(**a**) NEMA 17 in a CNC machine, (**b**) Flexible cable carriers, (**c**) DC motor, and (**d**) shaft.

The ability to print PCBs on copper surfaces has been achieved through the successful design and implementation of the CNC machine. It is intended for small-scale applications in educational institutions and specific manufacturing. The machine’s building technique is straightforward, energy efficient, and inexpensive^20^. The CNC machine is operated by a computer that runs the CNC-Shield-V3 and Arduino Uno, which are linked to three motors and allow the machine to move in the X, Y, and Z directions (cf. Figure 1). The machine is tested using kiCAD software to design a simplified PCB circuit, FlatCAM to convert files to G-Code format, and Universal G-Code Sender, a motor control program, to control and operate the machine. This allows the best performance out of the CNC machine, see Fig. 1.

### Controllers and electronics

To facilitate unrestricted movement of the CNC machine along the X, Y, and Z axes, the implementation of a NEMA 17 stepper motor is paramount, as substantiated by prior literature (see Fig. 1a,b)^20–22^. The NEMA 17 motor, a prominent variant of stepper motors, has been selected due to its exceptional capability to operate effectively in scenarios necessitating precise positioning and rapid response times. This motor exhibits a current rating of up to 2.5 A, thereby ensuring robust performance in demanding control applications^21–23^. The operational control of this prototype is managed by an Arduino UNO microcontroller, which employs software specifically designed to regulate the positioning of the stepper motors^24‚25^. Notably, the Arduino UNO distinguishes itself from other microcontroller boards by its omission of a USB-to-serial FTDI driver chip, thereby simplifying its integration. Built upon the ATmega328 microprocessor architecture, the Arduino UNO is equipped with 14 digital pins and six analog inputs, and it supports pulse-width modulation (PWM) functionality. The Arduino CNC Shield V3, an operating system tailored for this board, is explicitly designed to facilitate the construction of CNC machines across various applications, including drilling. This shield can accommodate multiple digital inputs and can control up to four DC motors, four stepper motors, and one servo motor^26^. Central to the operation of the NEMA 17 stepper motor is the DRV8825 motor driver, a high-current controller that serves as the intermediary between the Arduino microcontroller and the motor. The selection of the DRV8825 is predicated on its ability to interface seamlessly with both 3.3 V and 5 V logic levels, alongside its capacity to control bipolar stepper motors with an output current of up to 2.2 A per coil, negating the necessity for an additional logic voltage source. Furthermore, the DRV8825 driver offers high micro-stepping precision, achieving resolutions of up to 1/32 micro-stepping. This capability enables the precise control of the stepper motors, effectively reducing noise and vibrations, which is critical for producing smooth and accurate prints.

In the context of CNC operations, the configuration of three stepper motors—each responsible for movement along the X, Y, and Z axes—ensures the stability and precision required for the fabrication of intricate printed circuit structures. Given the demands of this application, the CNC machine’s framework must be robust, rigid, and meticulously designed to mitigate any oscillations or vibrations that could compromise accuracy during operation. To this end, the structure is crafted from aluminum, which guarantees high rigidity and durability, thereby enhancing the quality of the printing process. Additionally, the strategic placement of a wooden sheet atop the CNC machine bed serves to further stabilize the workpiece, ensuring optimal performance and precision during machining operations. Figures 1c,d. In the absence of wooden sheets atop the CNC machine’s bed, the exposed spindle, being inherently sharp, poses a significant risk of damaging the aluminum substrate. Conversely, should the spindle sustain damage or breakage during operation, the wooden sheets function as a sacrificial layer, thereby safeguarding the integrity of the CNC machine’s bed from the detrimental effects associated with cutting implements. This protective mechanism is essential for maintaining the longevity and operational efficiency of the equipment. It is especially crucial when making through-cuts, as the hardwood sheet will be sliced rather than the machine bed, preserving its original state. The wooden sheets are meticulously manufactured by measuring the dimensions of the bed, after which the laser cutting machine precisely cuts the wooden board to conform to these specifications.

There are four 3D-printed bases used to hold the CNC machine. They are one of the most important components as without those stands or bases, the CNC machine will suffer from oscillations and vibrations during printing on the printed circuit board that comes from the operation of the stepper motors and the DC motors, so using it ensures minimizing the overall vibrations and ensuring the stability of the CNC which lead due increasing the overall efficiency and accuracy during printing very small track widths. The chassis is an essential component in designing and operating CNC machines. It integrates all the electrical and mechanical components using polished and crossed nails to support the framework, component integration, and mechanical strength. Its sturdy aluminum structure and effective design substantially impact performance, dependability, and lifespan in various CNC machines where accuracy and stability are critical for operation. Within CNC machines, effective cable management is essential for ensuring the orderly arrangement, organization, and routing of electrical cables. Such measures are crucial to secure the cables firmly in place during machine operation, thereby preventing tangling and minimizing the potential for damage caused by abrasion or mechanical stress. In the context of this project, a flexible cable carrier has been implemented to systematically organize the electrical cables from the four stepper and DC motors.

The stepper motors, which are presented as cylindrical rods in CNC machines, play a pivotal role in transmitting rotational motion from one part of the machine to another. For instance, when the components of a CNC machine engage in operation—specifically during the printing phase—the shaft associated with these motors initiates rotation, thereby facilitating the free movement of the spindle within the machine’s framework. Interconnecting two shafts at their termini, a coupling device is employed to enhance operational efficiency. While torque-limiting couplings can disengage when the prescribed torque threshold is surpassed, conventional couplings serve the primary function of connecting two revolving components, thereby ensuring continuous operation^27^. In the present project, the coupler is fabricated from a rigid material optimized for CNC applications. Previous iterations utilized copper for this component, which was found to be inadequate for such use, primarily due to its compliance under mechanical stress and propensity to induce oscillations during CNC operation. These oscillations detract from the machine’s overall performance and lead to significant reductions in precision.

The V-bit tool is particularly notable in CNC applications, specifically for engraving tasks on printed circuit boards. Characterized by its pointed tip that allows for intricate and precise cutting, the V-bit tool exhibits a V-shaped geometry with angles that typically range from 10 to 30 degrees. The selection of V-bit dimensions is contingent upon the intended depth of cut, and these tools are available in a variety of materials to suit different applications. In contrast, the end mill differentiates itself from the drill bit; the latter is a cylindrical tool crafted solely for the purpose of creating holes in printed circuit boards through axial cutting. Conversely, the end mill is designed to perform cutting operations in all directions—both axial and radial. Furthermore, the drilling end mill represents a hybrid form that combines characteristics of both tools; it features a cylindrical shape akin to a drill bit, paired with a pointed tip reminiscent of an end mill. While drilling operations are performed axially, milling actions extend radially, allowing for a diverse range of cutting capabilities in CNC machining.

### Calibration

Calibrating a CNC machine is vital for PCB drilling as it aids in achieving the desired final product. Step/mm calibration is carried out for software calibration to ensure the CNC machine moves correctly based on the distance inserted in the software. In step/mm calibration, steps are the rotations made by the stepper motor in the CNC machine, while millimeters are the units of distance used to define the movements. When providing the CNC machine’s software information to move to a specific location, the software converts the distance required to cover into the number of steps the machine needs to take on the x, y, and z-axis. If the steps/mm value is inaccurate, the distance the drill bit covers might be less or more than desired. Due to this inaccuracy, the drilled holes on the PCB will be inaccurate in the long run. In this case, step/mm calibration is used by inputting the actual distance covered, which in return provides the user with the number of steps needed for the drill to move the required location. The user then inputs the correct number of steps for the drill to move to the correct point. A vernier caliper adds accurate measurements to the software when measuring the drill’s initial distance.

For accurate measurements, repetition of taking the readings is important to improve accuracy. In addition, the CNC machine should operate at room temperature to avoid thermal expansion. In general, the electrical components of the CNC machine contribute significantly to providing functionality for the machine. Multiple electrical components aid in this, such as the power supply that gives the components their required voltage and current. Controllers receive the G-code program and convert them into signals to the other components, such as the spindle and stepper motor drivers. These drivers control the motor by regulating the speed and torque based on the software’s drilling parameters. Another component in CNC machines is limiting switches, which are added to the axis of the machine to act as safety devices. When triggered, they signal the controller to stop moving the drill bit to prevent it from interfering with other machine components.

When stepper motors operate, the coils generate heat due to electrical resistance. In return, this degrades the motor bearings, shortening the lifespan and reducing performance. To counter this issue, the DRV8825 is an integrated circuit that is added to regulate the motor temperature. There are two methods that the DRV8825 works, the first of which is by limiting the maximum current that can flow through the motor windings. Although this can reduce the motor’s torque, it also lowers the heat produced by the motor, which reduces the chances of the motor getting damaged. Another adjustment that DRV8825 makes is that it can divide a full step by the motor into smaller steps; this also goes by micro-stepping. More micro steps mean smoother operation, but this increases current consumption.

There are some general steps involved in calibrating the DRV8825. Initially, the user should check the DRV8825 datasheet to understand the calibration process and the function of the control pins on the chip. After that, an initial run on the motor will be performed when it is operating, and the temperature will be measured using a non-contact thermometer. Next, begin with a low current limit and increase it gradually while checking on the motor’s temperature and performance until the desired torque is reached with a minimal current limit that does not produce much heat on the motor. Finally, try different micro-stepping modes until a balance between smooth motion, motor temperature, and current consumption is acquired.

By calibrating the DRV8825, the motor temperature can significantly decrease while maintaining performance and efficiency. Furthermore, the components of the CNC machine will last longer and have a lower risk of complete failure. Even though software calibration is crucial, hardware calibration is as important as software calibration. A main example of hardware calibration is spindling runout, which is the imperfection of the machine’s spindle. Normally, spindles should center perfectly on their axis, but this is usually not the case as the spindle wobbles slightly during rotation. Some of the reasons behind this can be the components worn from previous uses, manufacturing tolerances, or even incorrect part assembly. The reasons mentioned would then lead to radial and axial runout issues. Radial runout is when the center of the drill deviates from the circle, resulting in a hole diameter that is larger than the drill bit, which will cause the parts not to fit together correctly or lead to a wobbling motion. Axial runout is less common than radial motion but is as big of an issue as it is. In this case, the spindle axis is tilted compared to the ideal axis. This means that the spindle does not interact with the workpiece at a 90-degree angle, resulting in uneven drilling depths and increased wear and tear of the drill bit and its spindle due to uneven stresses applied to the tools.

When checking for spindle runout, it is advisable to use either a dial gauge or a test bar, depending on the type of runout. Dial gauges are normally used for radial runout by measuring the side-to-side movement of the drill bit holder, while test bars are used for axial runout by measuring the distance between the bar and the machine table at multiple points. Although spindle runout is a common issue, it does not have a definite solution to eliminate it. However, some precautions can be taken to ensure that its effect is minimal. These include performing regular maintenance of the machine spindle to reduce wear and tear or adjusting the hole size in your software to account for a known amount of radial runout. This method might not be ideal as it leads to inconsistencies. If the CNC machine is put in a state of disuse or infrequent use for a long time, then its rod’s flexibility and mobility will decrease, leading to several problems. One main factor leading to such deterioration is dust, debris, and other deposits building up on the rod and within the system’s working parts. These particles can rub against each other, interfering with the rod’s ease of movement. Also, if there is inadequate oiling or greasing, the fluids useful for the smooth running of the rod may dry out. This can lead to the stiffening of the rod over time and a general reduction in the tool’s flexibility.

Furthermore, external conditions such as humidity and temperature variations can cause corrosion and wear on the rod and other machine components. Metal components can deteriorate in high-humidity settings, developing rust and severely reducing rod movement. Similarly, temperature changes can cause metal parts to expand and contract, potentially resulting in misalignment and other mobility difficulties. Implementing a regular maintenance program is critical for mitigating these issues. This involves properly cleaning the machine to eliminate any collected debris, using proper lubricants on the rod and other moving elements, and storing the equipment in a stable, climate-controlled environment. Regularly checking the equipment for signs of wear and tear can also help recognize and resolve issues before they become major concerns. By correctly maintaining the CNC machine, you can keep the rod flexible and movable, extending the machine’s life and performance.

## AI based PCB design

**Fig. 2:**
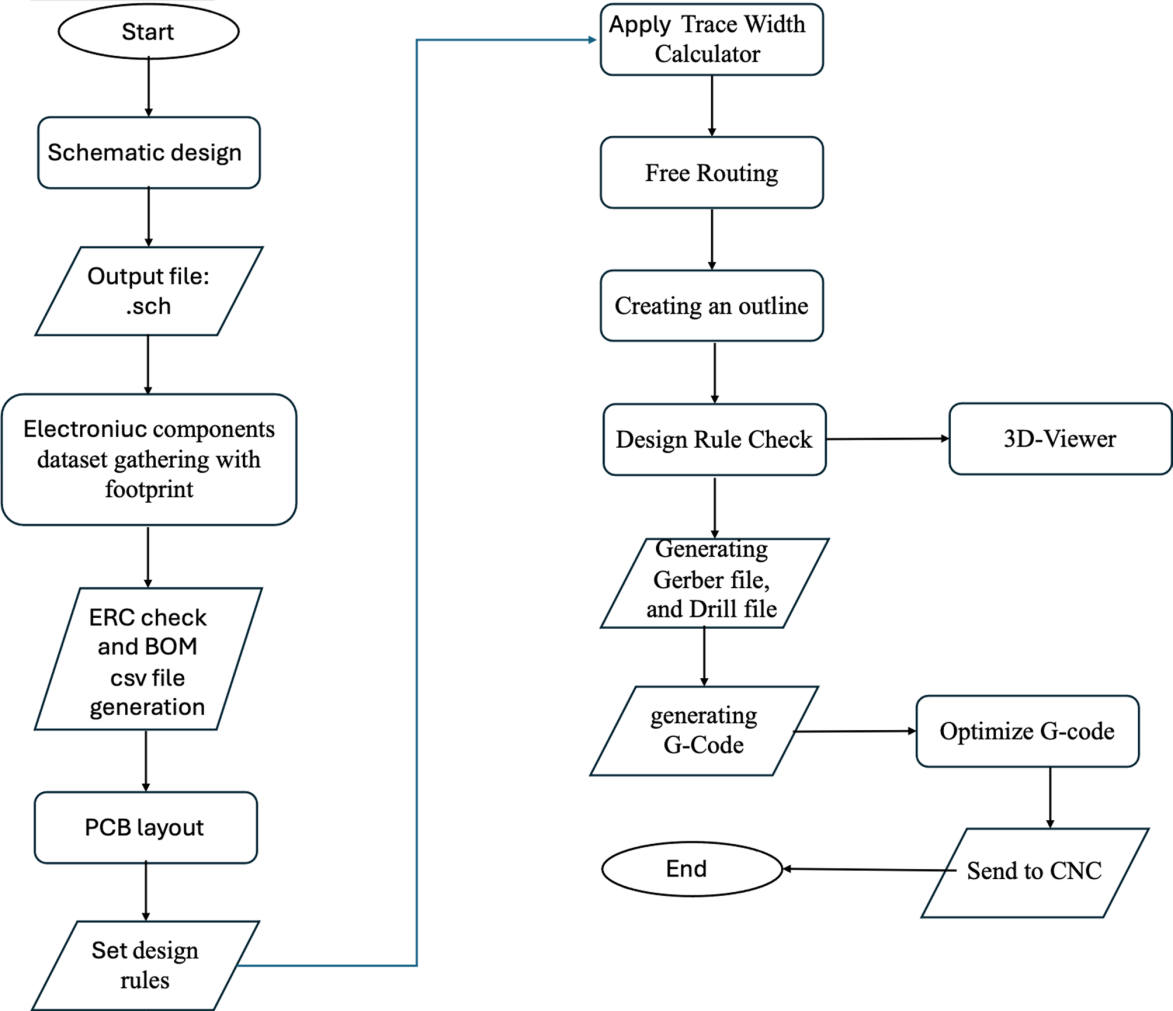
PCB design flowchart.

The procedure for designing and implementing a circuit on a PCB involves three steps to generate the executable file that the CNC machine can read to start printing tracks and drilling holes. In brief, the first step is to design the schematic on an EDA tool and construct the PCB layout. Then, extract the Gerber files for different layers. The third step is to read the Gerber files and construct the g-Code (aka CNC job file); see the flowchart in Fig. 2. This section explains the detailed procedure for each step. A script is developed to automate these processes using PowerShell scripting to facilitate the job of an electronic circuit designer and automate the repetitive processes.

### First stage: schematic design

**Fig. 3:**
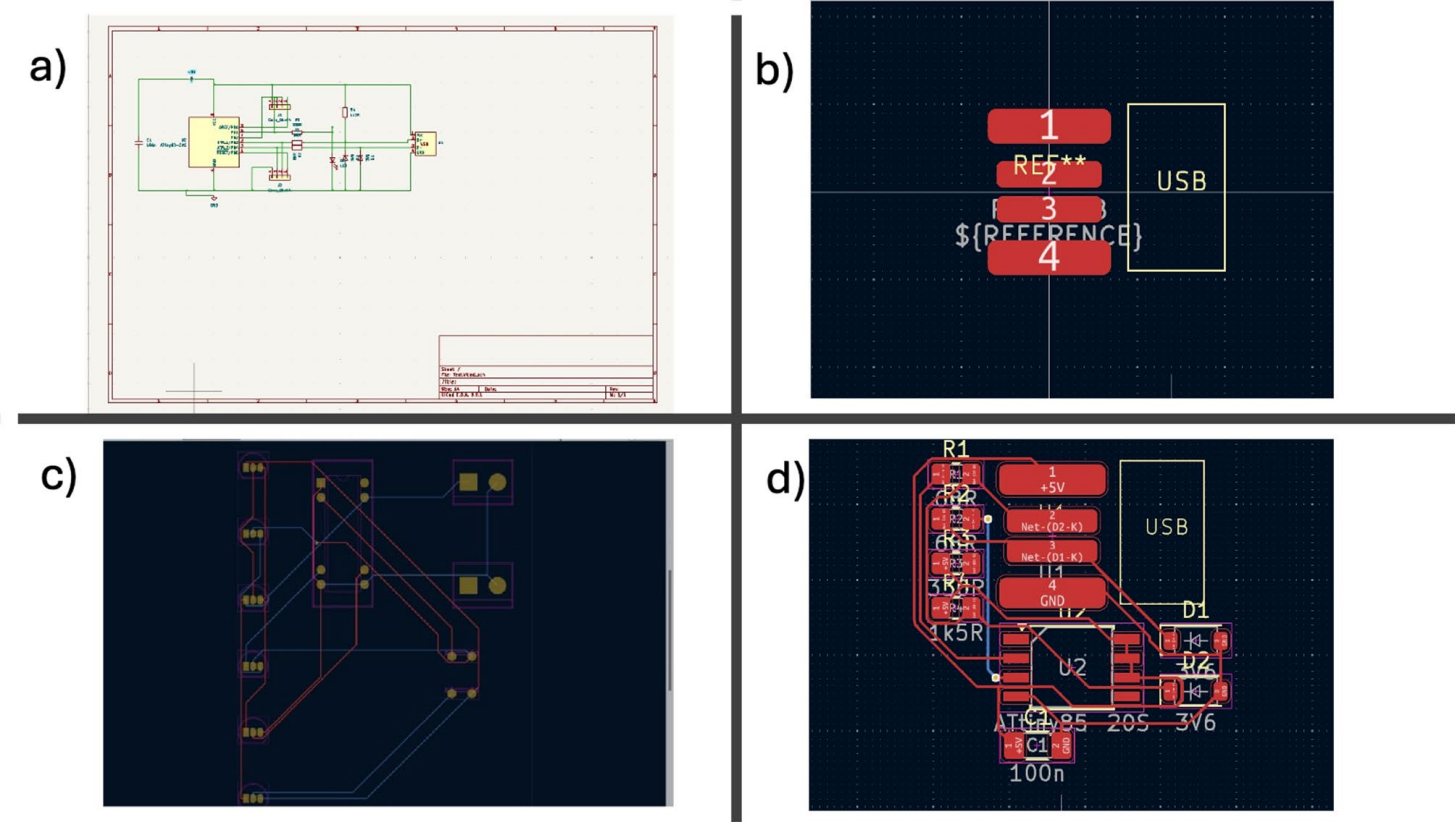
(**a**) Schematic Editor of KiCad, (**b**) Component placed in footprint editor, (**c**) Example of Free Routing, and (**d**) Converting schematic into physical PCB layout.

The initial step in our process involves the schematic design of the circuit using a CAD tool capable of exporting a.sch file. In this study, we utilized KiCad’s Schematic Editor, as illustrated in Fig. 3a. The KiCad Scripting Console represents a significant advancement in Electronic Design Automation (EDA), as it not only automates repetitive tasks but also enhances the functionality of KiCad by enabling the generation of custom reports and the development of new tools. Accessible from both the Schematic Editor and PCB Editor, the scripting console provides a robust Python API that allows users to manipulate KiCad projects, schematics, PCBs, and libraries. The KiCad Scripting Console can automate various repetitive tasks, including component placement, netlist generation, and design rule checks. Furthermore, it supports the creation of custom scripts tailored for specific purposes. In the context of this project, several use cases for the Scripting Console have been developed and will be briefly outlined. Initially, it is necessary to install the required Python modules and libraries for the script to function effectively.

Among the most critical modules within the KiCad Scripting Console is **pcbnew**, which encompasses all functions available in the KiCad PCB editor, thereby facilitating the automation of repetitive tasks and enabling features that are not achievable through manual processes. For instance, the following snippet reads the current board, identifies its components or modules by their reference designators, adjusts their positions relative to the origin, and updates the PCB view accordingly. Additionally, it generates the netlist and conducts Design Rule Checks (DRC) for tracks narrower than 2 mm, issuing a warning message to the user. Moreover, this project incorporates a function to generate a PDF report from a Bill of Materials (BOM) CSV file produced by KiCad, supplemented with additional data such as maximum current ratings for active components (e.g., transistors and amplifiers), orientation, positioning, and availability of components within Egypt, where our laboratory is situated. A comprehensive dataset comprising components such as BJTs, FETs, amplifiers, gates, and comparators has been developed for this project and is utilized as a reference for determining track widths based on input currents. This dataset is aligned with the components available in stock to ensure consistency and reliability. It is also associated with the footprint of each electronic component, which defines the arrangement of holes or pads required to connect electronic components with the specified shape on a PCB, as depicted in Fig. 3b. The footprint is designed to be compatible with the physical dimensions of the components.

Furthermore, for variants of electronic components not available in KiCad, users have the option to create them using the Symbol Editor and define their footprints using the Footprint Editor. The schematic design can be verified through the Electrical Rules Check (ERC), which identifies issues such as unconnected pins, missing power connections, and short circuits. While the ERC cannot guarantee the overall functionality of the schematic, it provides valuable insights into potential problems that may arise. Subsequently, a Bill of Materials (BOM) is generated in a CSV file for all components utilized in the schematic design. The netlist defines the relationships between the electronic components and their corresponding footprints and must be regenerated whenever the schematic is modified. Most netlists include or reference details of the parts or devices used, with each part referred to as an instance. These descriptions typically encompass a list of the connections made to each type of device, along with essential attributes of the devices. The connecting points are commonly referred to as terminals or pins. The generation of the netlist concludes the first stage of our process. In addition, the integration of machine learning techniques into this framework could further enhance the design and optimization processes. By employing machine learning algorithms, we can analyze historical design data to predict optimal drilling paths, improve component placement strategies, and refine the overall design process based on performance metrics. This approach allows for adaptive learning, enabling the system to continuously improve its recommendations and efficiency, ultimately leading to superior outcomes in PCB manufacturing.

### Second step: PCB layout

The PCB Editor in KiCad is a comprehensive tool for designing printed circuit boards. It allows users to convert schematic designs into physical PCB layouts, enabling the creation of electronic prototypes and products. The user interface is like and as simple as the schematic editor, which contains a workspace to design and arrange PCB components and tracks. It differs in that it has Layer Management that controls multiple layers for component placement, routing, silkscreen, and solder mask. The design rules menu sets rules for tracks, such as track width, minimum clearance, minimum connection width, and other parameters for holes and vias. These parameters must be tested before using the CNC machine used to design the board. The user can set different track widths from the predefined sizes tab and assign them to different Net classes.

Herein, we develop a Trace Width Calculator (TWC), a tool to calculate the trace width of a PCB using a random forest regression model. Determining trace width depends on several factors, such as maximum current, temperature, and power dissipation. IPC standards recommend track widths for one (oz/square feet) copper PCB at a 10o temperature rise. In the current context, the trace width ($\:w$) is detrained as:

1 $$\:w=\frac{I}{k.{\left(T\right)}^{b}}.({h)}^{c}$$

where $\:T$ is the temperature rise above ambient in degrees Celsius (°C), $\:I$ is the current in amperes, $\:h$ is the trace thickness in mils (for example, 1 oz/ft² copper is about 1.4 mils thick), while $\:k$, $\:b$, and $\:c$ are constants that depend on whether the trace is on the outer or inner layers of the PCB.

Importing Netlists brings all footprints and interconnections placed previously on the Schematic editor to the PCB Editor. After Adjusting the PCB Layout and moving components in place, the next process is routing. Various routing techniques have been introduced previously in the literature^14,28,29^. Manual hand routing is an option, although it is recommended that free routing is used in sophisticated circuits. Free routing is added to KiCAD Plugins as an external plugin. The earlier rules, such as track width, clearance width, and vias rules, will be used to autoroute traces in Free Routing. After using Free Routing (Fig. 3c), the user can manually adjust some routes or change a 45-angle track to a circular one.

**Fig. 4:**
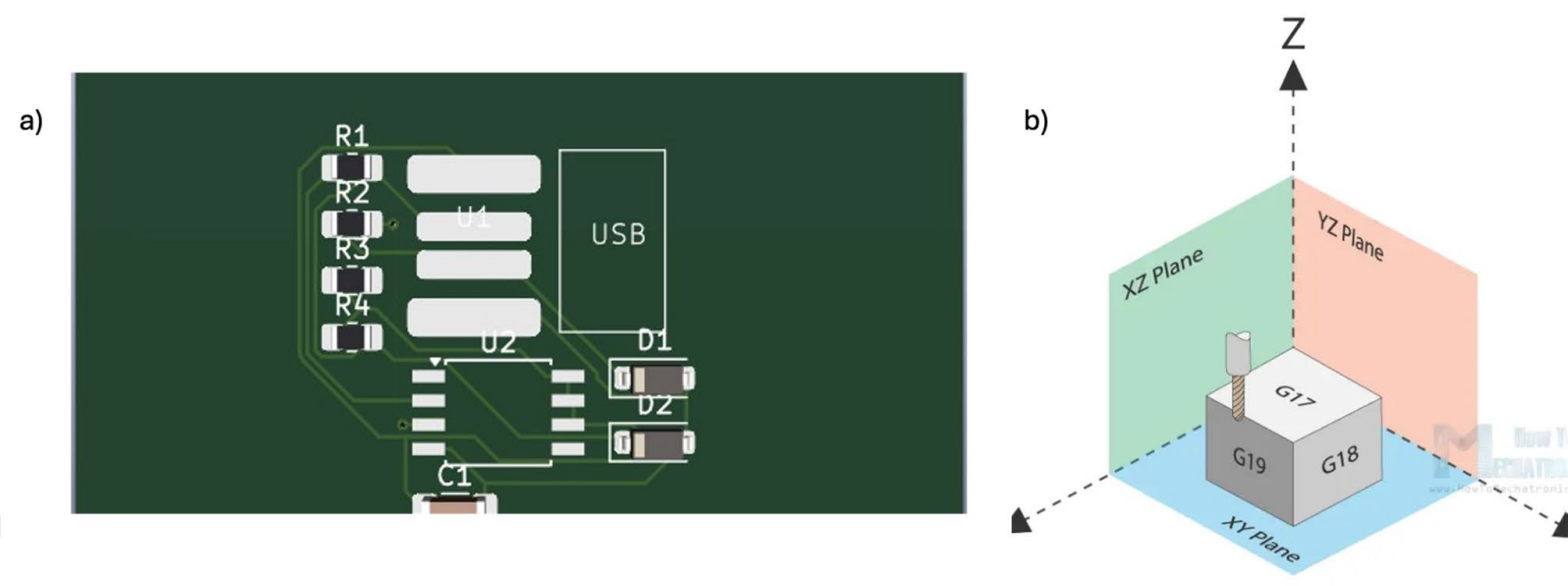
(**a**) Front copper layer in 3D viewer, and (**b**) G18 for XZ and G19 for YZ plane.

Creating an outline for the board is crucial since it determines the size of the board, cf. Figure 3d. For a production board, choose the minimum available size to use the board piece, while there is no restriction on experimental and educational purposes. Creating an outline is done through the edge cuts layer. Then, running Design Rule Check (DRC) to check for errors is important to reduce the probability of errors in the design. Running DRC is like ERC explained previously. Track errors appear with an arrow to indicate a design rule violation. Tracks like the ones with arrows need to be adjusted manually by a technician. The design rule violation may be due to misconfigured parameters set in the design rule settings (e.g., small maximum clearance). In addition, the 3D-Viewer can be used to show the PCB’s different layers and vias in 3D View or the front and back copper layers, as in Fig. 4a. Finally, using a plot icon, Gerber files can be generated to describe copper positioning on a PCB. Then, Gerber will select the layers that need to be generated. Gerber Files must be generated after all DRC checks and design rule violations are fixed. Generating drill files is much like generating Gerber files, with careful choice of the Units millimeter or inch depending on the design and the drill origin being the same origin as the Gerbers.

### Third step: generating G-CODE

Geometric Code, commonly referred to as G-code, constitutes a specialized set of instructions that govern machine movements in CNC systems and 3D printers across three axes. This coding format employs a variety of symbols to convey specific commands essential for precise operation. Upon obtaining the Gerber files, several steps are undertaken to extract the corresponding G-codes utilizing CAD software designed for PCB prototyping, such as FlatCAM. In this context, a straightforward Gerber file representing the Back Copper layer of a PCB serves as the basis for generating the G-code, as illustrated in Fig. 4b. The optimization of G-code to achieve an efficient code with minimal length and reduced travel time is of paramount importance in PCB design, particularly in the context of mass production. Most CAD software tools, including FlatCAM, incorporate a range of optimization algorithms aimed at generating the most efficient code possible. Nevertheless, the G-code can be further refined to align with specific machine capabilities and the requisite accuracy of the operation.

**Fig. 5:**
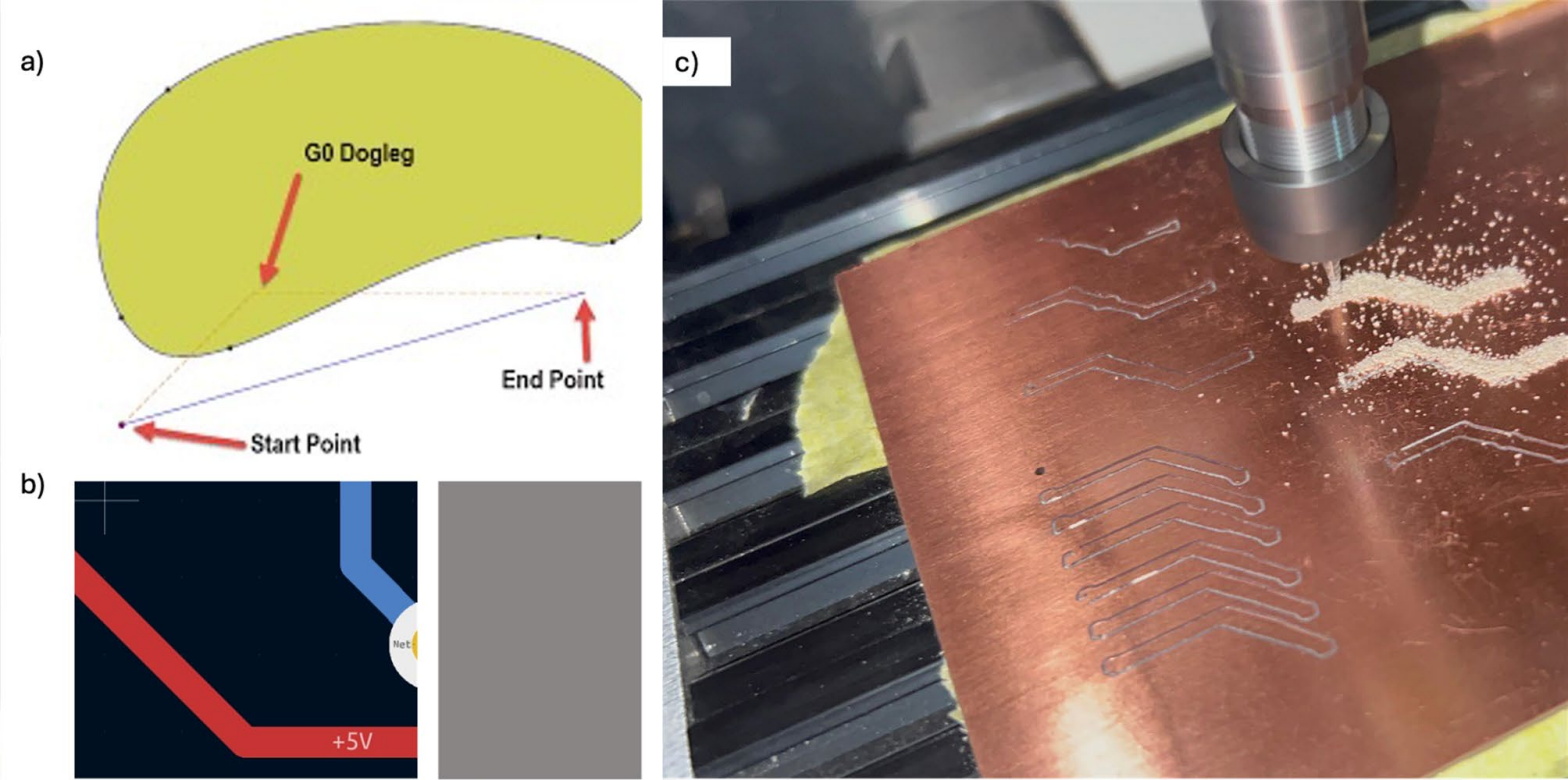
(**a**) Dogleg moves during printing, (**b**) Two straight lines connected with angle 135, and (**c**) Testing tracks using different feed rates.

Minimizing Toolpath Length: This objective can be realized by consolidating similar movements into fewer command lines. This approach not only optimizes the number of code lines (NOC) but also effectively reduces the overall length of the code. Reducing Non-Cutting Movements: Further optimization can be achieved by minimizing the non-cutting movements, specifically through the refinement of retracts and approaches. By selecting a minimum length for movements in the Z-direction, as previously detailed in the G-code explanation section, the upward movements of the tool after completing a cut can be significantly reduced. Optimizing Cutting Parameters: This optimization involves adjusting various feed rates tailored to different machining processes. Notably, the drilling feed rate is distinct from the XY feed rate; furthermore, the XY feed rate may vary depending on the specific process being executed, as demonstrated in Fig. 5. By fine-tuning these parameters, one can enhance the efficiency and precision of the machining operation, ultimately contributing to improved outcomes in PCB fabrication.

### Path planning and optimization

**Fig. 6:**
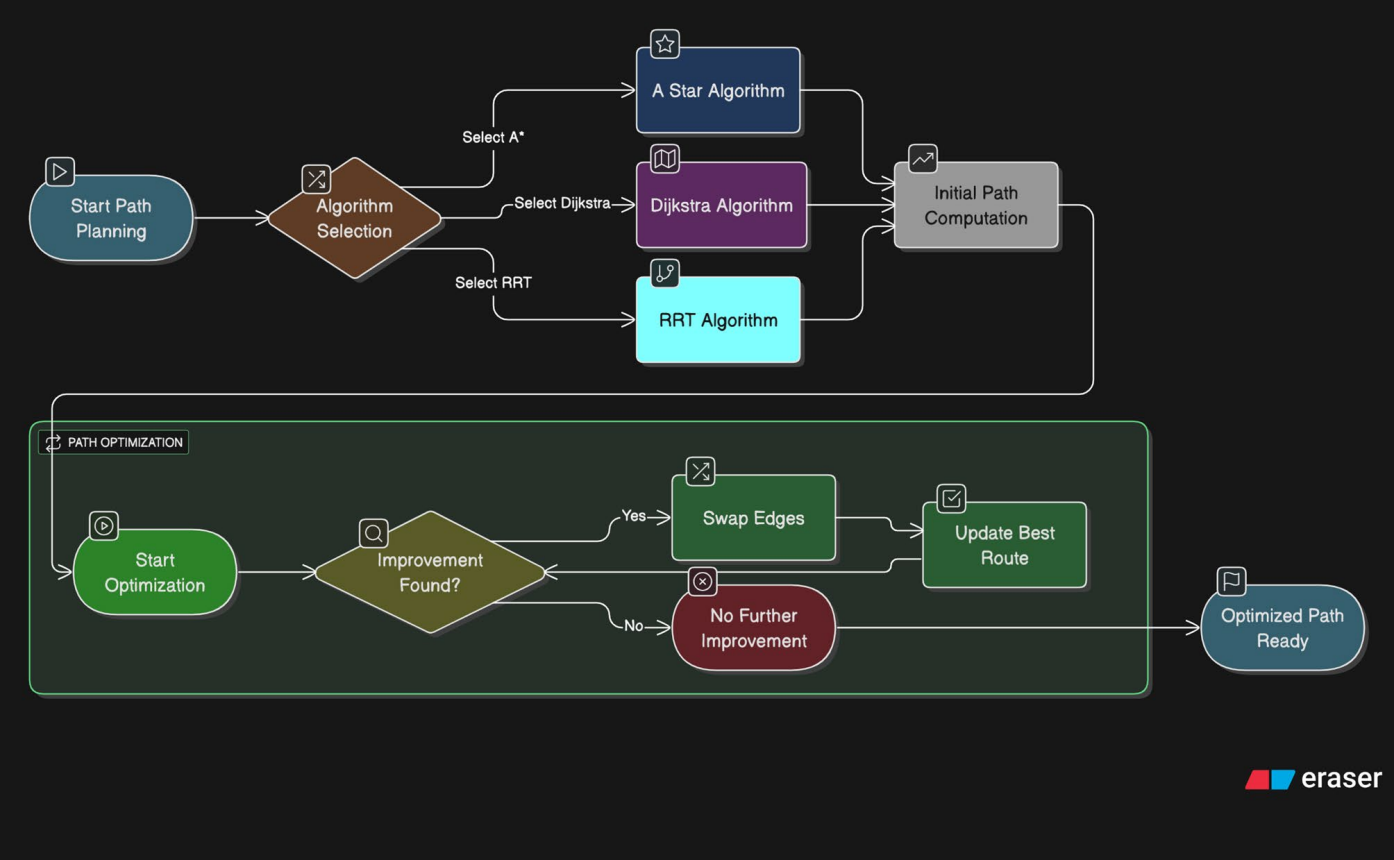
Path planning optimization algorithm.

Path planning and optimization are critical components in enhancing the efficiency and effectiveness of machining operations, particularly in the context of PCB manufacturing. The objective of path planning is to determine a viable trajectory for the tool to traverse, achieving the desired machining outcomes while optimizing this trajectory to minimize resource expenditures such as time and energy, see Fig. 6. Among the prevalent algorithms employed for path planning, the A* (A-star) algorithm, Dijkstra’s algorithm, and the Rapidly-exploring Random Tree (RRT) algorithm stand out for their efficacy in navigating complex environments. The A* algorithm utilizes a heuristic approach, balancing the cost of the path already traveled against an estimate of the cost to reach the destination. This is formulated as a cost function

2 $$\:f\left(n\right)=g\left(n\right)+h\left(n\right)$$

where $\:g\left(n\right)\:$represents the actual cost from the start node to node $\:n$, and $\:h\left(n\right)$ denotes the estimated cost from node $\:n$ to the goal. This combination facilitates the prioritization of nodes that appear to lead to the most efficient path. The algorithm systematically explores paths utilizing a priority queue, achieving a complexity class of $\:O\left(n\text{log}n\right)\:$where $\:n$ is the number of nodes in the pathfinding space. In contrast, Dijkstra’s algorithm guarantees the shortest path in weighted graphs by exploring all possible paths from the starting node to the destination. Although it ensures optimality, it lacks heuristic guidance, resulting in longer computational times that can be expressed as $\:O\left({n}^{2}\right)$for the standard implementation. This inefficiency becomes particularly evident in complex environments where numerous paths must be evaluated.

The RRT algorithm excels in high-dimensional spaces due to its probabilistic nature. It incrementally builds a tree by randomly selecting points in the space and extending the tree towards these points while ensuring obstacle avoidance, making it particularly suited for dynamic path planning tasks. To further improve the paths generated by these algorithms, iterative optimization techniques can be employed, such as the 2-opt algorithm. This technique systematically examines pairs of edges within the path to evaluate whether swapping them results in a shorter overall route. The basic logic can be outlined as follows: for each pair of edges $\:(A,\:B)$ in the path, the potential new path length is evaluated by calculating the total path length before and after performing the swap. The swap occurs if the new path length is shorter, thereby enhancing the path’s efficiency. The time complexity of the basic 2-opt algorithm is $\:O\left({n}^{2}\right)$, where $\:n\:$represents the number of edges in the path. However, it often leads to substantial improvements in practical scenarios where the number of edge swaps necessary to achieve optimality is significantly lower.

### Automated process

The Manual steps to create the schematic, design the PCB, apply routing algorithms, choose the best one for the design, generate Gerber files, and extract the geometry and G-code from Gerbers are cumbersome and have repetitive tasks. Automating these steps allows circuit designers `to focus on innovative tasks and reduce overhead on repetitive tasks. Repeating the steps explained earlier times is cumbersome and repetitive. Thus, an automated TCL script has been developed to read Gerbers, process them, and generate the G-Codes. However, the G-Codes generated from the script are not superior to the ones generated directly by the FlatCAM software and need optimization. This is true since FlatCAM applies optimization techniques in the manual process, and these optimization algorithms are embedded in it, starting with version 8.0.

Reading and generating G-Codes involves several key steps to ensure efficient operation. First, the directories for the input Gerber files and the output G-Code to be generated must be established. Next, essential physical parameters, including spindle rate, z-cut depth, feed rate in both the $\:XY$ and $\:Z$ directions and the layers to be processed, need to be configured. Following this setup, the Gerber files are read to extract the necessary design information. Finally, the G-Code is generated from the imported Gerber files, facilitating precise instructions for the drilling machine’s operation. The two G-Codes generated by the automated and manual processes show the travel distance and estimated time using the same parameters as those of the CNC machine. The G-code length in the automated process is 7181 lines, while in the manual process is 4776 lines.

Furthermore, a designer must be familiar with all the software tools like KiCAD (Schematic Editor, PCB Editor, and Console Editor), FlatCAM, and Universal G-Code Editor. However, this knowledge could be minimized to only the Schematic editor and Universal g-Code editor by automating the creation of the PCB, Automatic placement in PCB view, generating Gerber files, and extracting the g-codes in FlatCAM. Two scripts were created using PowerShell scripting and Python to do the mentioned tasks. The PowerShell script uses built-in functions to Send Keyboard Keys and mouse clicks. Another imprint feature is related to the current abilities of components. Finding the Maximum Current of a component is done by creating a dataset of the components used in KiCAD, such as BJTs, FETs, Amplifiers, and comparators. A Dataset of more than 1800 components is created. It is being updated with max current and availability in Egypt to allow for better settings for trace width depending on current input and choice or an alternative component if it is not present in Egypt. However, the latter part is a recommendation and hasn’t been applied.

Gerber Files and drill files are generated using two separate functions for each. Hole coordinates and diameter are set in the script for the drill file, while for Gerbers, specific layers are generated. A failed trial for generating G-codes directly from Gerbers is worth mentioning in this context. However, it tried to read the Gerber, extract coordinates, and determine the G-Code operation (G00, G01, etc.) to write it in the cnc_job file. Eventually, the Python script automates the previously mentioned KiCAD processes, and the creation of G-Codes using FlatCAM by running the TCL mentioned earlier in this context. Python script to automate these tasks has proved more reliable than PowerShell script.

## AI based PCB design

This section demonstrates the testing phase to ensure the robustness of the design, as well as design-processing and experimental tests extracted from the current study.

### Testing clearance and track width

**Fig. 7:**
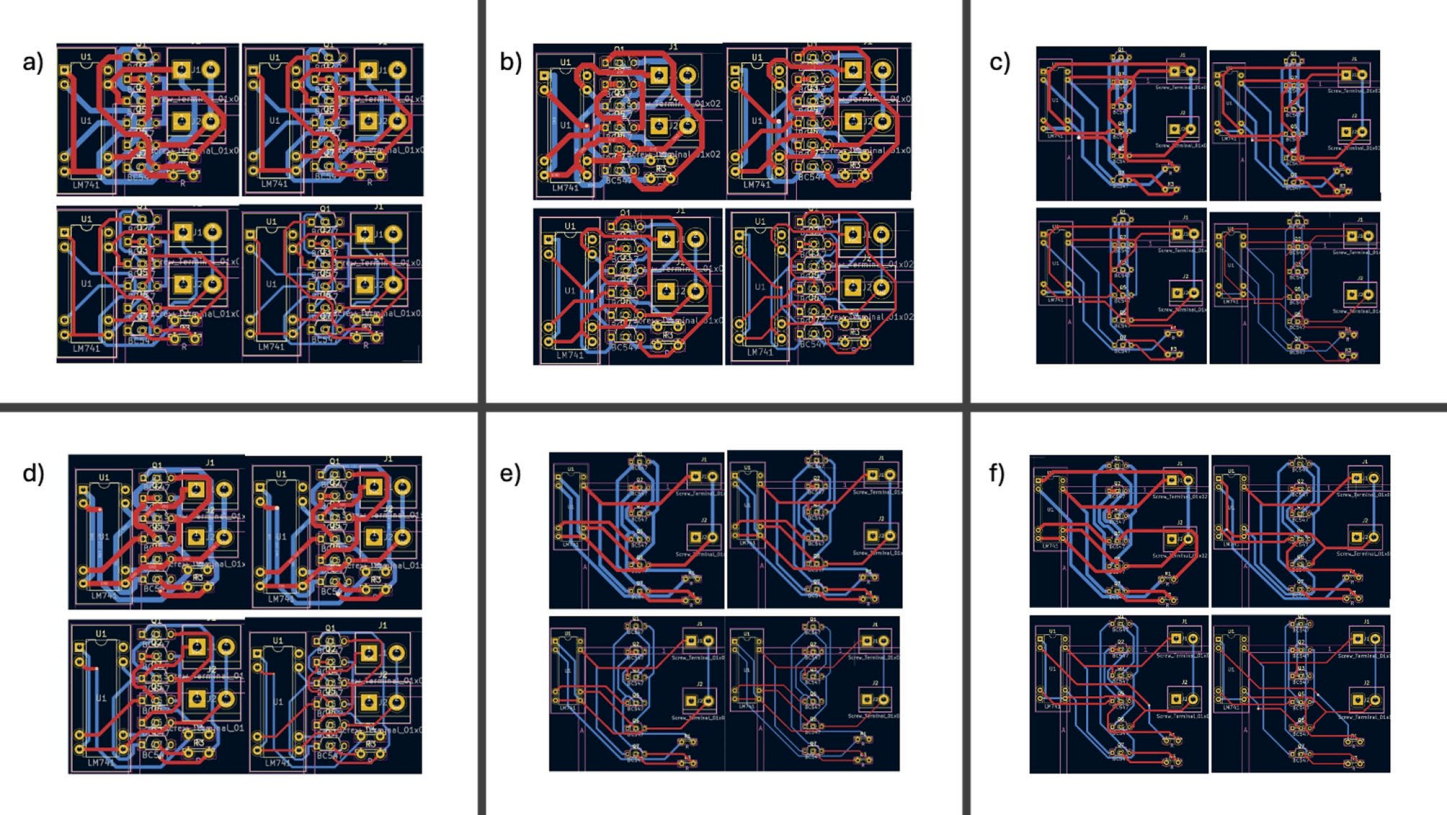
Six PCB layouts featured as: (**a**) non-spaced PCB layout with 0.5 mm clearance, (**b**) spaced PCB layout with 0.5 mm clearance, (**c**) non-spaced PCB layout with 0.35 mm clearance, (**d**) spaced PCB layout with 0.35 mm clearance, (**e**) non-spaced PCB layout with 0.2 mm clearance and (**f**) spaced PCB layout with 0.2 mm clearance.

Clearance and track width contributes heavily in the operation of auto routing, the track width is an important parameter in this operation as typically a matching between the width and current is advertised to reduce the possibility of power loss, ensuring a adequate clearance is also important usually high clearance is preferred in the concept of PCB manufacturing which help to neglect hardware issues, as if tracks are too close to each other a hardware issue could lead to interconnection between tracks, on the counterpart increasing the clearance will give a challenging task to autoroutes and then increase the possibility that the design cannot be fulfilled or huge processing power is used so a very crucial study is to test differences of perquisite clearance values (0.5,0.35,0.2) clearance over different track width which ranges from 1 mm to 0.4 mm with a step size of 0.2 mm, additionally a software test is carried to indicate the processing power needed if circuit components increase, cf. Figure 7-a.

**Fig. 8:**
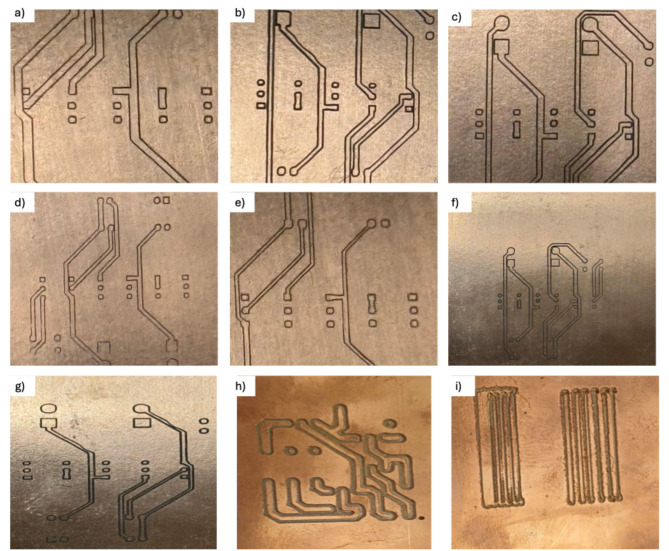
(**a**) 0.6 mm width and 0.5 mm clearance, (**b**) 0.8 mm width and 0.5 mm clearance, (**c**) 1 mm width and 0.5 mm clearance, (**d**) 0.4 mm width and 0.5 mm clearance, (**e**) 0.6 mm and 0.35 mm clearance, (**f**) 1 mm width and 0.35 mm clearance, (**g**) 0.4 mm width and 0.35 mm clearance, (**h**) overlapping tracks, and (**i**) testing different width.

Another approach added to this test involves introducing more spacing between components, which optimizes auto-routing efficiency and helps with different clearances. Starting with 0.5 mm clearance, a PCB can be routed without increasing the space between components, and the time needed for auto-routing is recorded (see Fig. 7-b to f). The data presented in Table 1 illustrate a direct correlation between processing time and clearance; as clearance increases, processing time also increases. Conversely, the relationship with width demonstrates a distinct trend, as an increase in width enhances the performance of the auto-router. Notably, if the number of circuit components increases significantly, the processing time can escalate dramatically, potentially rendering it impossible for the auto-router to function effectively. Furthermore, the outputs depicted in Fig. 8 present a series of tested layouts with varying clearances and widths, aimed at evaluating the operational capacity of the CNC machine. This experimentation records the processing times of the auto-router to elucidate the relationships among processing time, clearance, width, and overall hardware performance.

**Table 1:** Testing processing time over different width and clearance size with spaced and unspaced components, experimentally measured data based on our proposed prototype.

Width (mm)	Clearance (mm)	Processing time(spaced/not spaced)
1 mm	0.5 mm	5 s/22 s
0.8 mm	0.5 mm	5 s/19 s
0.6 mm	0.5 mm	4 s/15 s
0.4 mm	0.5 mm	4 s/9 s
1 mm	0.35 mm	3 s/17 s
0.8 mm	0.35 mm	2 s/13 s
0.6 mm	0.35 mm	2 s/12 s
0.4 mm	0.35 mm	2 s/9 s
1 mm	0.2 mm	5 s/16 s
0.8 mm	0.2 mm	3 s/13 s
0.6 mm	0.2 mm	2 s/5 s
0.4 mm	0.2 mm	2 s/3 secondds

### Case study for routing in low power DC-DC converter

**Fig. 9:**
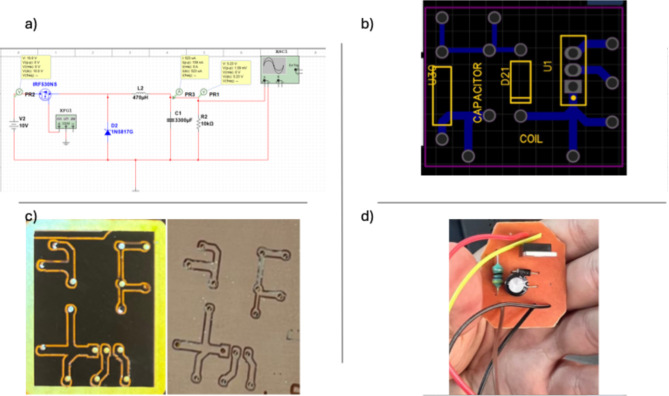
(**a**) Circuit schematic created in KiCad, illustrating a 10 V voltage supply, a MOSFET acting as a switch (IRF530NS), a 1N5817G diode, a 470 µH inductor paired with a 3300 µF capacitor, and a 10 kΩ resistor, (**b**) PCB layout of the circuit, (**c**) the circuit printed on the PCB following the drilling process, and (**d**) soldering of circuit components onto the PCB.

To verify the appropriate development of the software cycle and the hardware implementation, we have tested the designed prototype among a wide range of electronic circuits with variations in operation condition, DC or AC, as well as in the maximum power. One of these attempts was a low-power Buck converter, which bucks the voltage from 10 V to 5.23 V. Typically, the application of this circuit is to be used for energy harvesting accompanied by 14 Piezoelectrical cells to drive a small load, as illustrated in Fig. 9a. The circuit then proceeded to be drawn in PCB layout mode after choosing the suitable footprint that applies to our application (Fig. 9b); afterward, the routing is done (Fig. 9c). The circuit is optimized in 30 cm by 24 cm (this measurement is for the Borders) and then processed to be executed by the following software, which generates the G-code.

FlatCAM is used as it provides an intuitive interface that allows users to import Gerber files and drill files, edit them, and generate the necessary toolpaths for PCB manufacturing. It also has the cutting-edge feature of choosing a suitable Milling tip, applying different values of clearance and feed rate, and even performing isolation routing. After the circuit has been created, it’s a CNC job, and the output is shown below. To verify the operationality, the first test uses the 0.1 mm 15-degree milling tip in the first trial and showcases some printing deficiencies, which include lousy finishing of the tracks (However, it still passes the continuity test), typically this is done as the clearance of the tracks is selected to be minimum as possible to ensure high output quality, then a 0.2 mm 45-degree milling tip is used and showcases an optimized path with better output and improved track finishing, see Fig. 9d. The change is quite noticeable, making FLATCAM a good match for a notable prediction on the milling tip. After finishing the PCB layout, a continuity test was done through all the tracks to verify its operationality.

### Performance comparison and experimental validation

To substantiate the efficacy of our proposed path planning and optimization model, a comprehensive performance comparison was conducted against existing commercial and academic solutions, including KiCAD’s native auto-router and the optimization settings of FlatCAM, as well as other G-code generators. The baseline for this comparison was established using standard routing configurations from KiCAD, which serves as a widely recognized benchmark in the PCB design community. Under controlled conditions, we evaluated routing efficiency by measuring key performance metrics such as processing time, tolerance accuracy, drill misalignment rates, and time-to-finish for fabrication. These metrics were systematically recorded across multiple test boards to ensure robust experimental validation. Our model demonstrated a remarkable 13-fold improvement in routing efficiency when compared to the baseline established by KiCAD’s auto-router. This assertion is grounded in empirical data collected under consistent testing conditions, where the same PCB designs were subjected to both our optimized routing algorithm and the KiCAD native solution. Measurements were meticulously taken to quantify processing times, with specific attention to the impact of various width and clearance sizes, as illustrated in Table 1.

The experimental data reveal that processing times significantly decreased for both spaced and unspaced components, with notable improvements in efficiency as the width and clearance were optimized. For instance, at a width of 0.4 mm and a clearance of 0.35 mm, our model achieved a processing time of merely 2 s for spaced components, compared to 9 s for the same configuration using the KiCAD auto-router. Such results underscore the effectiveness of our approach in minimizing fabrication time while maintaining stringent accuracy standards. To further validate our findings, we conducted a statistical performance analysis encompassing error margins, standard deviations, and success rates across multiple test boards. This analysis revealed that our model consistently achieved tolerance accuracy within ± 0.05 mm, with a drill misalignment rate of less than 2% across the various configurations tested. The success rates were notably high, with over 95% of the test boards meeting the predefined design specifications. These quantitative measures not only reinforce the validity of our performance claims but also highlight the potential of our prototype to significantly enhance manufacturing efficiency in PCB production. In summary, the comparative analysis and experimental validation presented herein demonstrate that our proposed model not only surpasses existing solutions in terms of routing efficiency but also provides a statistically robust framework for assessing performance metrics critical to high-quality PCB fabrication.

## Conclusion

Introducing AI in circuit design comes with great benefits in terms of accuracy, innovation, and efficiency. Using AI in PCB design and manufacturing processes has reduced the required time to complete design and generate Gerber files and G-Codes compared to manual methods. Moreover, the skill level needed to complete these tasks requires highly skilled engineers and technicians, and in some cases, requires a whole team to complete the design and manufacture of a single PCB. However, this project uses a collection of scripts to automate the mentioned processes after the circuit schematic is completed.

KiCAD EDA software tool creates schematics and PCBs and generates Gerber files. A dataset comprising BJTs, FETs, amplifiers, and many more electronic components is used in this project, along with their maximum input current and availability in the country. The minimum track width is automatically determined based on the dataset. The placement of components in the PCB area after the circuit schematic is completed is done automatically, along with routing using free routing. A case study comparing manual routing with auto-routing is carried out here. It shows that auto routing via the Free Routing plugin in KiCAD improves the average time required to complete routing by more than 13 times compared to manual routing. Furthermore, Gerber and drill files are generated automatically by leveraging the KiCAD scripting console.

Processing Gerber files to generate G-Codes is done using FlatCAM software. Generating multiple G-codes from multiple Gerber files is automated to save time using a TCL script. A continuity test is done after a PCB is created and the PCB functionality for the first time. A test case was carried out in two trials to verify the operation of the PCB at the British University in Egypt, using two milling tips of 0.1 mm with 15 degrees in the first trial and 0.2 mm with 45 45-degree milling tips in the second trial. The second test produced a higher-quality finish than the first trial, which had printing deficiencies. However, both trials passed the continuity test.

## Data Availability

The data supporting this study’s findings are available from the corresponding author upon reasonable request.
